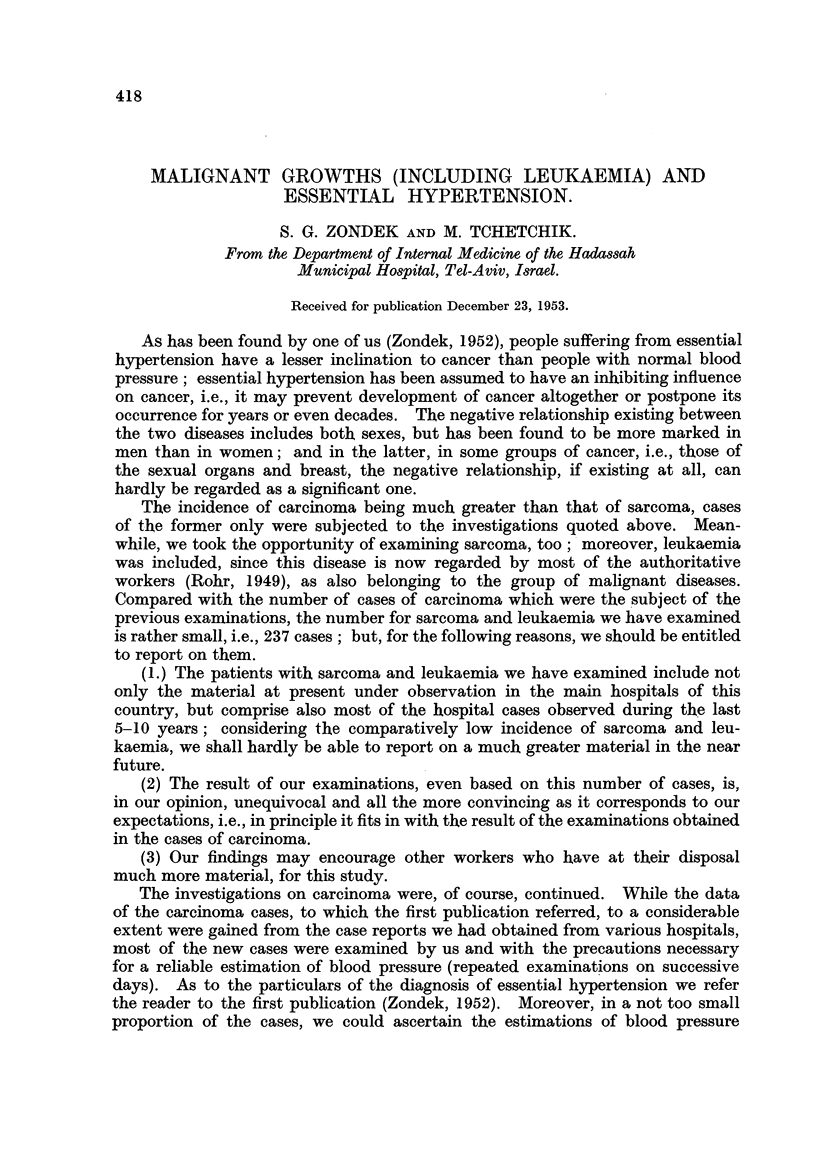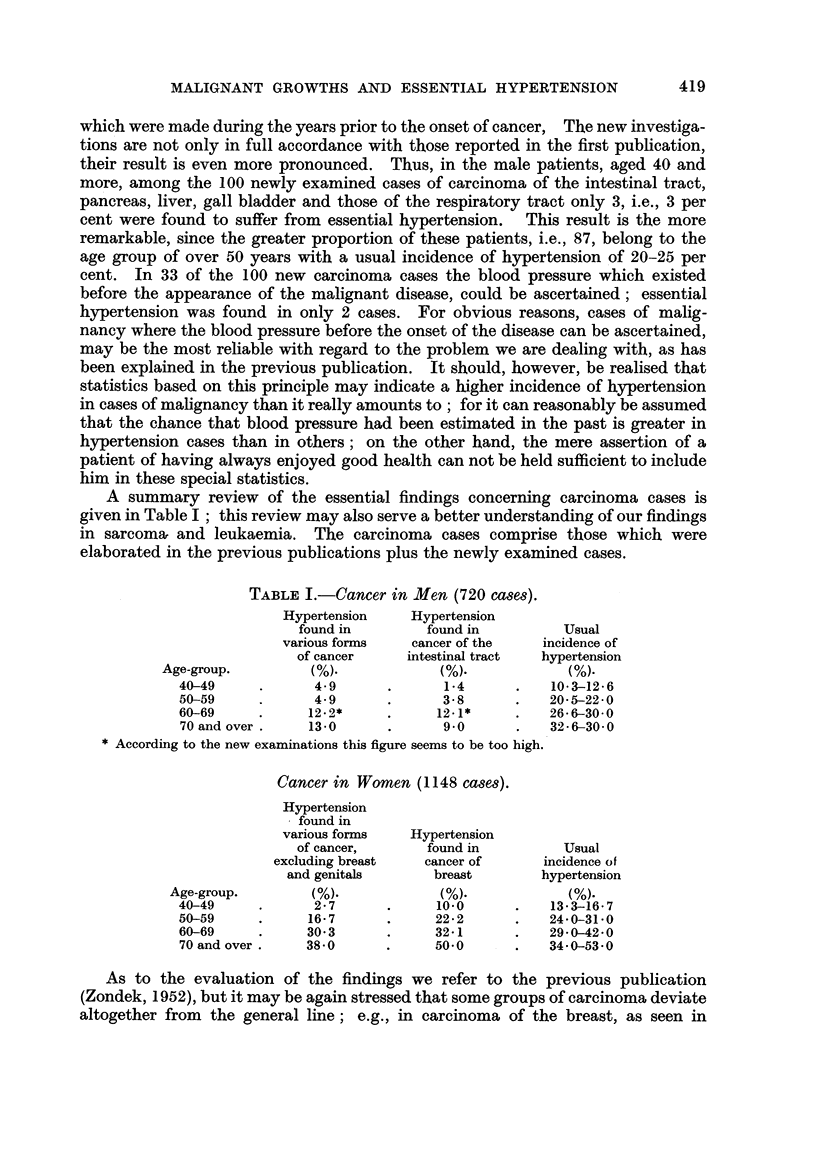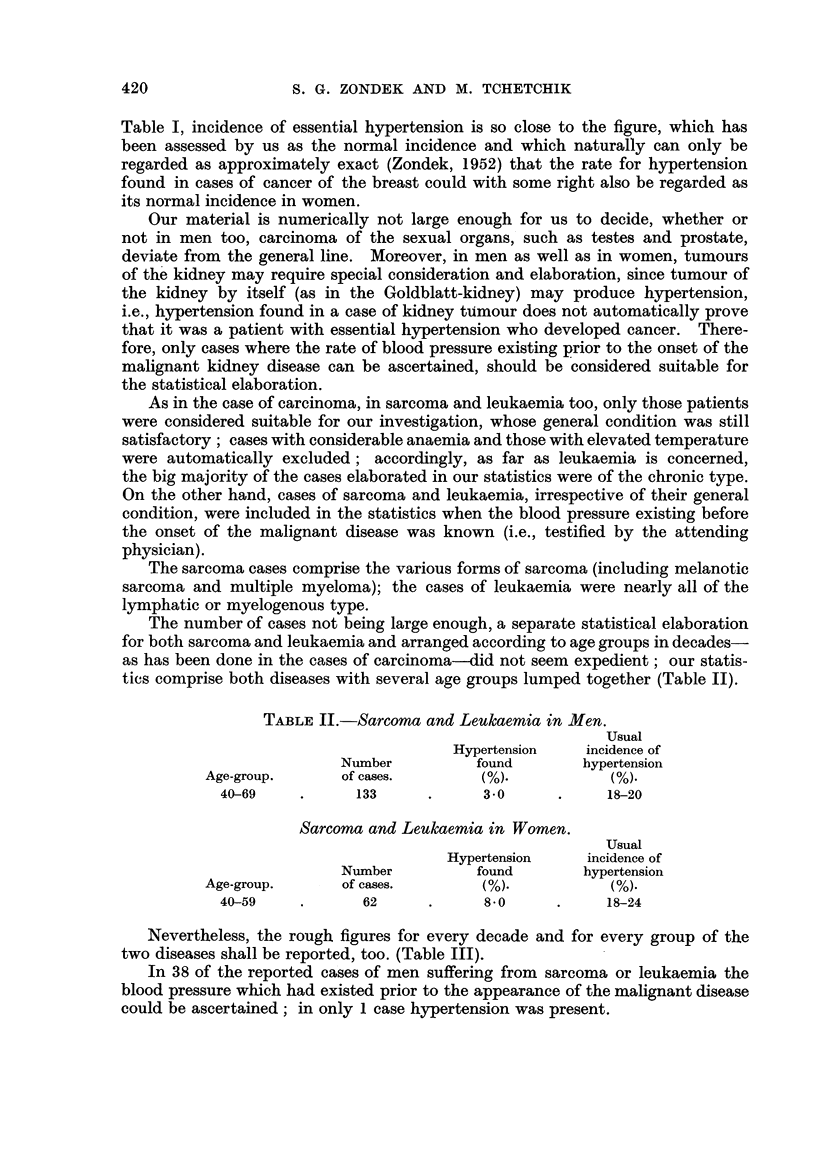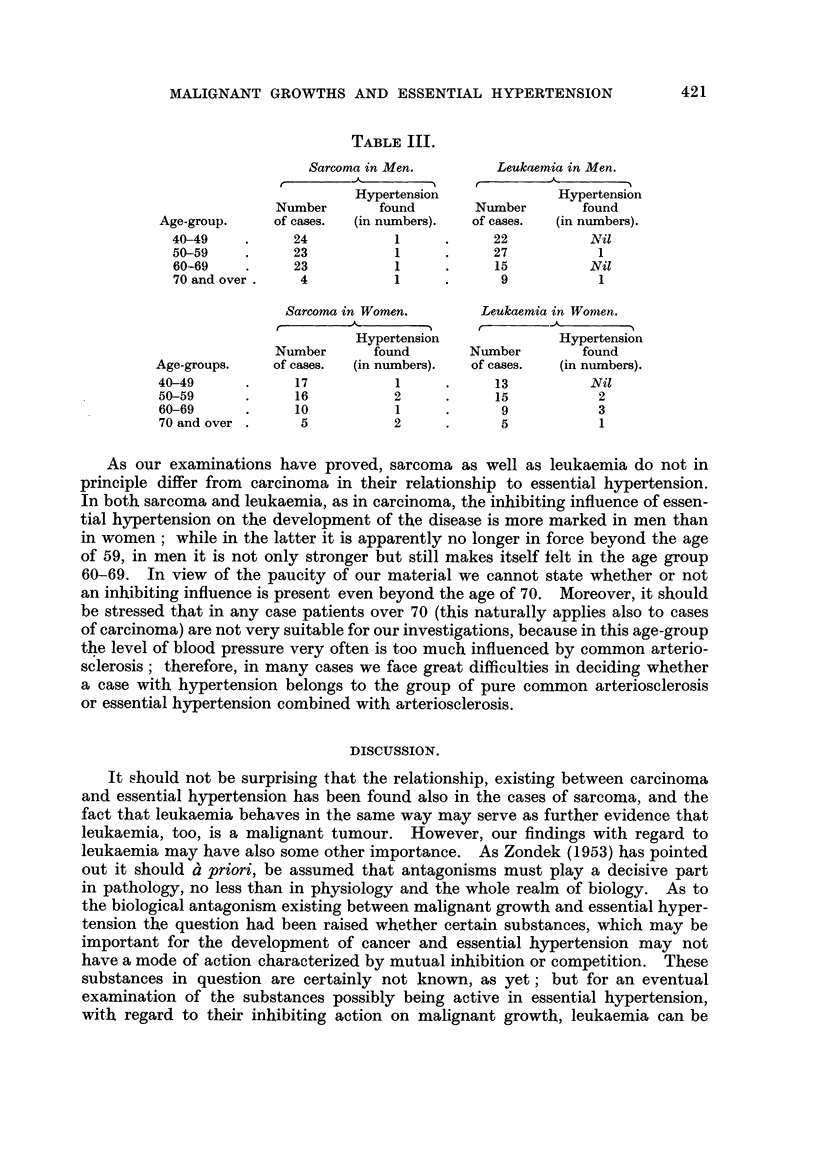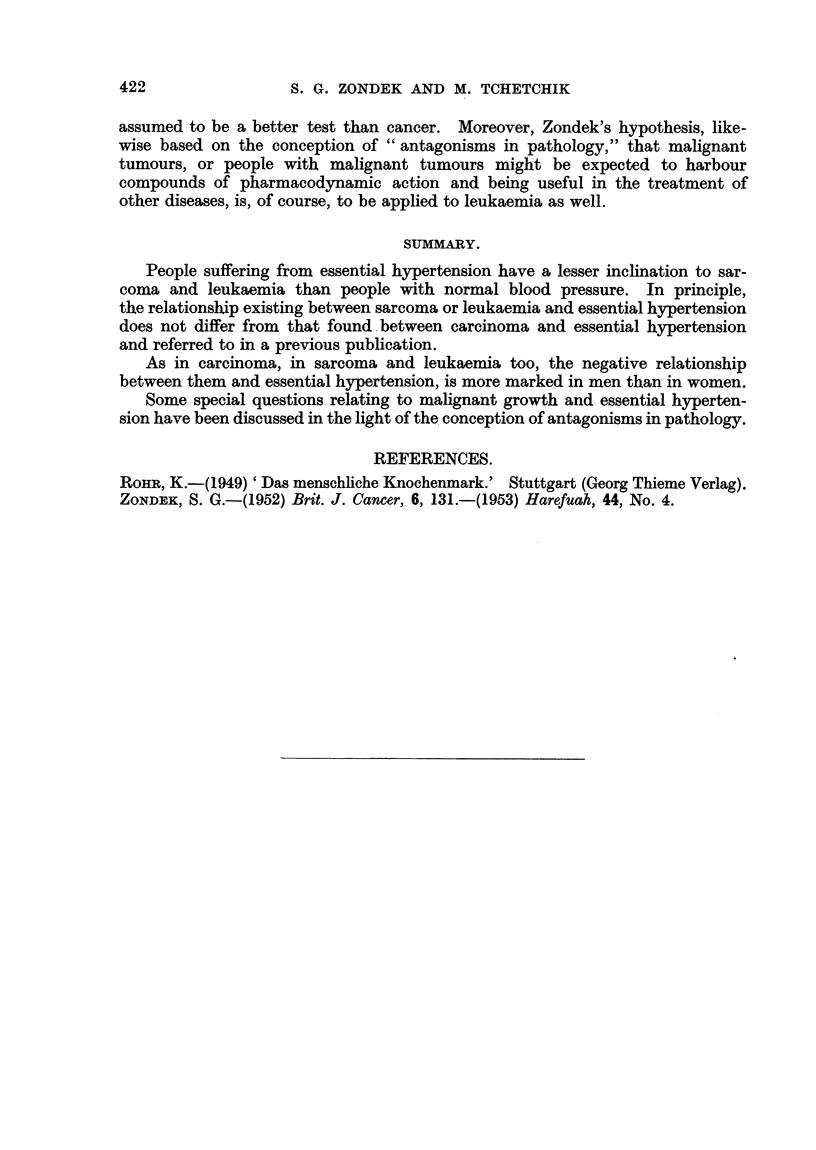# Malignant Growths (including Leukaemia) and Essential Hypertension

**DOI:** 10.1038/bjc.1953.40

**Published:** 1953-12

**Authors:** S. G. Zondek, M. Tchetchik


### MALIGNANT GROWTHS (INCLUDING LEUKAEMIA) AND ESSENTIAL HYPERTENSION.

S. G. ZONDEK AND M. TCHETCHIK.

*From the Department of Internal Medicine of the Hadassah Municipal Hospital, Tel-Aviv, Israel.*

Received for publication December 23, 1953.

As has been found by one of us (Zondek, 1952), people suffering from essential hypertension have a lesser inclination to cancer than people with normal blood pressure ; essential hypertension has been assumed to have an inhibiting influence on cancer, i.e., it may prevent development of cancer altogether or postpone its occurrence for years or even decades. The negative relationship existing between the two diseases includes both sexes, but has been found to be more marked in men than in women; and in the latter, in some groups of cancer, i.e., those of the sexual organs and breast, the negative relationship, if existing at all, can hardly be regarded as a significant one.

The incidence of carcinoma being much greater than that of sarcoma, cases of the former only were subjected to the investigations quoted above. Meanwhile, we took the opportunity of examining sarcoma, too ; moreover, leukaemia was included, since this disease is now regarded by most of the authoritative workers (Rohr, 1949), as also belonging to the group of malignant diseases. Compared with the number of cases of carcinoma which were the subject of the previous examinations, the number for sarcoma and leukaemia we have examined is rather small, i.e., 237 cases ; but, for the following reasons, we should be entitled to report on them.

(1.) The patients with sarcoma and leukaemia we have examined include not only the material at present under observation in the main hospitals of this country, but comprise also most of the hospital cases observed during the last 5–10 years ; considering the comparatively low incidence of sarcoma and leukaemia, we shall hardly be able to report on a much greater material in the near future.

(2) The result of our examinations, even based on this number of cases, is, in our opinion, unequivocal and all the more convincing as it corresponds to our expectations, i.e., in principle it fits in with the result of the examinations obtained in the cases of carcinoma.

(3) Our findings may encourage other workers who have at their disposal much more material, for this study.

The investigations on carcinoma were, of course, continued. While the data of the carcinoma cases, to which the first publication referred, to a considerable extent were gained from the case reports we had obtained from various hospitals, most of the new cases were examined by us and with the precautions necessary for a reliable estimation of blood pressure (repeated examinations on successive days). As to the particulars of the diagnosis of essential hypertension we refer the reader to the first publication (Zondek, 1952). Moreover, in a not too small proportion of the cases, we could ascertain the estimations of blood pressure which were made during the years prior to the onset of cancer, The new investigations are not only in full accordance with those reported in the first publication, their result is even more pronounced. Thus, in the male patients, aged 40 and more, among the 100 newly examined cases of carcinoma of the intestinal tract, pancreas, liver, gall bladder and those of the respiratory tract only 3, i.e., 3 per cent were found to suffer from essential hypertension. This result is the more remarkable, since the greater proportion of these patients, i.e., 87, belong to the age group of over 50 years with a usual incidence of hypertension of 20–25 per cent. In 33 of the 100 new carcinoma cases the blood pressure which existed before the appearance of the malignant disease, could be ascertained ; essential hypertension was found in only 2 cases. For obvious reasons, cases of malignancy where the blood pressure before the onset of the disease can be ascertained, may be the most reliable with regard to the problem we are dealing with, as has been explained in the previous publication. It should, however, be realised that statistics based on this principle may indicate a higher incidence of hypertension in cases of malignancy than it really amounts to ; for it can reasonably be assumed that the chance that blood pressure had been estimated in the past is greater in hypertension cases than in others ; on the other hand, the mere assertion of a patient of having always enjoyed good health can not be held sufficient to include him in these special statistics.

A summary review of the essential findings concerning carcinoma cases is given in Table I ; this review may also serve a better understanding of our findings in sarcoma and leukaemia. The carcinoma cases comprise those which were elaborated in the previous publications plus the newly examined cases.

TABLE I.—*Cancer in Men* (720 *cases*).

| Age-group. | Hypertension found in various forms of cancer (%). | Hypertension found in cancer of the intestinal tract (%). | Usual incidence of hypertension (%). |
|---|---|---|---|
| 40–49 | 4·9 | 1·4 | 10·3–12·6 |
| 50–59 | 4·9 | 3·8 | 20·5–22·0 |
| 60–69 | 12·2* | 12·1* | 26·6–30·0 |
| 70 and over | 13·0 | 9·0 | 32·6–30·0 |

* According to the new examinations this figure seems to be too high.

*Cancer in Women* (1148 *cases*).

| Age-group. | Hypertension found in various forms of cancer, excluding breast and genitals (%). | Hypertension found in cancer of breast (%). | Usual incidence of hypertension (%). |
|---|---|---|---|
| 40–49 | 2·7 | 10·0 | 13·3–16·7 |
| 50–59 | 16·7 | 22·2 | 24·0–31·0 |
| 60–69 | 30·3 | 32·1 | 29·0–42·0 |
| 70 and over | 38·0 | 50·0 | 34·0–53·0 |

As to the evaluation of the findings we refer to the previous publication (Zondek, 1952), but it may be again stressed that some groups of carcinoma deviate altogether from the general line ; e.g., in carcinoma of the breast, as seen in

Table I, incidence of essential hypertension is so close to the figure, which has been assessed by us as the normal incidence and which naturally can only be regarded as approximately exact (Zondek, 1952) that the rate for hypertension found in cases of cancer of the breast could with some right also be regarded as its normal incidence in women.

Our material is numerically not large enough for us to decide, whether or not in men too, carcinoma of the sexual organs, such as testes and prostate, deviate from the general line. Moreover, in men as well as in women, tumours of the kidney may require special consideration and elaboration, since tumour of the kidney by itself (as in the Goldblatt-kidney) may produce hypertension, i.e., hypertension found in a case of kidney tumour does not automatically prove that it was a patient with essential hypertension who developed cancer. Therefore, only cases where the rate of blood pressure existing prior to the onset of the malignant kidney disease can be ascertained, should be considered suitable for the statistical elaboration.

As in the case of carcinoma, in sarcoma and leukaemia too, only those patients were considered suitable for our investigation, whose general condition was still satisfactory ; cases with considerable anaemia and those with elevated temperature were automatically excluded ; accordingly, as far as leukaemia is concerned, the big majority of the cases elaborated in our statistics were of the chronic type. On the other hand, cases of sarcoma and leukaemia, irrespective of their general condition, were included in the statistics when the blood pressure existing before the onset of the malignant disease was known (i.e., testified by the attending physician).

The sarcoma cases comprise the various forms of sarcoma (including melanotic sarcoma and multiple myeloma); the cases of leukaemia were nearly all of the lymphatic or myelogenous type.

The number of cases not being large enough, a separate statistical elaboration for both sarcoma and leukaemia and arranged according to age groups in decades—as has been done in the cases of carcinoma—did not seem expedient ; our statistics comprise both diseases with several age groups lumped together (Table II).

TABLE II.—*Sarcoma and Leukaemia in Men.*

| Age-group. | Number of cases. | Hypertension found (%). | Usual incidence of hypertension (%). |
|---|---|---|---|
| 40–69 | 133 | 3·0 | 18–20 |

*Sarcoma and Leukaemia in Women.*

| Age-group. | Number of cases. | Hypertension found (%). | Usual incidence of hypertension (%). |
|---|---|---|---|
| 40–59 | 62 | 8·0 | 18–24 |

Nevertheless, the rough figures for every decade and for every group of the two diseases shall be reported, too. (Table III).

In 38 of the reported cases of men suffering from sarcoma or leukaemia the blood pressure which had existed prior to the appearance of the malignant disease could be ascertained ; in only 1 case hypertension was present.

TABLE III.

| Age-group. | Sarcoma in Men. Number of cases. | Sarcoma in Men. Hypertension found (in numbers). | Leukaemia in Men. Number of cases. | Leukaemia in Men. Hypertension found (in numbers). |
|---|---|---|---|---|
| 40–49 | 24 | 1 | 22 | *Nil* |
| 50–59 | 23 | 1 | 27 | 1 |
| 60–69 | 23 | 1 | 15 | *Nil* |
| 70 and over | 4 | 1 | 9 | 1 |

| Age-groups. | Sarcoma in Women. Number of cases. | Sarcoma in Women. Hypertension found (in numbers). | Leukaemia in Women. Number of cases. | Leukaemia in Women. Hypertension found (in numbers). |
|---|---|---|---|---|
| 40–49 | 17 | 1 | 13 | *Nil* |
| 50–59 | 16 | 2 | 15 | 2 |
| 60–69 | 10 | 1 | 9 | 3 |
| 70 and over | 5 | 2 | 5 | 1 |

As our examinations have proved, sarcoma as well as leukaemia do not in principle differ from carcinoma in their relationship to essential hypertension. In both sarcoma and leukaemia, as in carcinoma, the inhibiting influence of essential hypertension on the development of the disease is more marked in men than in women ; while in the latter it is apparently no longer in force beyond the age of 59, in men it is not only stronger but still makes itself felt in the age group 60–69. In view of the paucity of our material we cannot state whether or not an inhibiting influence is present even beyond the age of 70. Moreover, it should be stressed that in any case patients over 70 (this naturally applies also to cases of carcinoma) are not very suitable for our investigations, because in this age-group the level of blood pressure very often is too much influenced by common arteriosclerosis ; therefore, in many cases we face great difficulties in deciding whether a case with hypertension belongs to the group of pure common arteriosclerosis or essential hypertension combined with arteriosclerosis.

### DISCUSSION.

It should not be surprising that the relationship, existing between carcinoma and essential hypertension has been found also in the cases of sarcoma, and the fact that leukaemia behaves in the same way may serve as further evidence that leukaemia, too, is a malignant tumour. However, our findings with regard to leukaemia may have also some other importance. As Zondek (1953) has pointed out it should *à priori*, be assumed that antagonisms must play a decisive part in pathology, no less than in physiology and the whole realm of biology. As to the biological antagonism existing between malignant growth and essential hypertension the question had been raised whether certain substances, which may be important for the development of cancer and essential hypertension may not have a mode of action characterized by mutual inhibition or competition. These substances in question are certainly not known, as yet ; but for an eventual examination of the substances possibly being active in essential hypertension, with regard to their inhibiting action on malignant growth, leukaemia can be assumed to be a better test than cancer. Moreover, Zondek's hypothesis, likewise based on the conception of " antagonisms in pathology," that malignant tumours, or people with malignant tumours might be expected to harbour compounds of pharmacodynamic action and being useful in the treatment of other diseases, is, of course, to be applied to leukaemia as well.

### SUMMARY.

People suffering from essential hypertension have a lesser inclination to sarcoma and leukaemia than people with normal blood pressure. In principle, the relationship existing between sarcoma or leukaemia and essential hypertension does not differ from that found between carcinoma and essential hypertension and referred to in a previous publication.

As in carcinoma, in sarcoma and leukaemia too, the negative relationship between them and essential hypertension, is more marked in men than in women.

Some special questions relating to malignant growth and essential hypertension have been discussed in the light of the conception of antagonisms in pathology.

### REFERENCES.

ROHR, K.—(1949) 'Das menschliche Knochenmark.' Stuttgart (Georg Thieme Verlag).

ZONDEK, S. G.—(1952) *Brit. J. Cancer*, **6**, 131.—(1953) *Harefuah*, **44**, No. 4.